# NeatFreq: reference-free data reduction and coverage normalization for *De Novo* sequence assembly

**DOI:** 10.1186/s12859-014-0357-3

**Published:** 2014-11-19

**Authors:** Jamison M McCorrison, Pratap Venepally, Indresh Singh, Derrick E Fouts, Roger S Lasken, Barbara A Methé

**Affiliations:** Informatics Core Services, The J. Craig Venter Institute (JCVI), 9704 Medical Center Drive, Rockville, MD 20850 USA; Department of Genomic Medicine, The J. Craig Venter Institute (JCVI), 9704 Medical Center Drive, Rockville, MD 20850 USA; Department of Microbial & Environmental Genomics, The J. Craig Venter Institute (JCVI), 9704 Medical Center Drive, Rockville, MD20850 USA

**Keywords:** Bioinformatics, *de novo* assembly, Coverage reduction, Normalization, Single cell, SISPA, Transcriptomics, Multiple displacement amplification

## Abstract

**Background:**

Deep shotgun sequencing on next generation sequencing (NGS) platforms has contributed significant amounts of data to enrich our understanding of genomes, transcriptomes, amplified single-cell genomes, and metagenomes. However, deep coverage variations in short-read data sets and high sequencing error rates of modern sequencers present new computational challenges in data interpretation, including mapping and *de novo* assembly. New lab techniques such as multiple displacement amplification (MDA) of single cells and sequence independent single primer amplification (SISPA) allow for sequencing of organisms that cannot be cultured, but generate highly variable coverage due to amplification biases.

**Results:**

Here we introduce NeatFreq, a software tool that reduces a data set to more uniform coverage by clustering and selecting from reads binned by their median kmer frequency (RMKF) and uniqueness. Previous algorithms normalize read coverage based on RMKF, but do not include methods for the preferred selection of (1) extremely low coverage regions produced by extremely variable sequencing of random-primed products and (2) 2-sided paired-end sequences. The algorithm increases the incorporation of the most unique, lowest coverage, segments of a genome using an error-corrected data set. NeatFreq was applied to bacterial, viral plaque, and single-cell sequencing data. The algorithm showed an increase in the rate at which the most unique reads in a genome were included in the assembled consensus while also reducing the count of duplicative and erroneous contigs (strings of high confidence overlaps) in the deliverable consensus. The results obtained from conventional Overlap-Layout-Consensus (OLC) were compared to simulated multi-de Bruijn graph assembly alternatives trained for variable coverage input using sequence before and after normalization of coverage. Coverage reduction was shown to increase processing speed and reduce memory requirements when using conventional bacterial assembly algorithms.

**Conclusions:**

The normalization of deep coverage spikes, which would otherwise inhibit consensus resolution, enables High Throughput Sequencing (HTS) assembly projects to consistently run to completion with existing assembly software. The NeatFreq software package is free, open source and available at https://github.com/bioh4x/NeatFreq.

**Electronic supplementary material:**

The online version of this article (doi:10.1186/s12859-014-0357-3) contains supplementary material, which is available to authorized users.

## Background

The multiple displacement amplification (MDA) reaction allows for single cell sequencing and genome assembly of organisms that cannot be cultured [1]. MDA is also frequently used to amplify DNA from low biomass environmental samples for use in metagenomic sequencing although amplification bias alters the ratio with which individual species are represented [2]. During shotgun sequencing, genomic libraries are randomly sampled from a population of molecules; this sampling is biased due to sample content and preparation. Such selection bias is even more prominent when MDA is used to amplify DNA from a single cell [3-5]. The amplified DNA has extreme coverage variability and may represent from only a small portion of the genome span up to the complete recovery of the genome [1]. Sequence independent single primer amplification (SISPA) allows for sequencing of organisms that cannot be cultured, including single cell bacterial genomes [4,6], viral genomes [7-9], and metagenomes [10-13]. Selection bias within SISPA-prepared sequences also results in extreme coverage variability. The biases in sequence coverage from both approaches lead to an increased probability that rarely occurring sequences will be removed when reads are selected randomly for the purpose of coverage reduction. Similar outcomes can also occur due to experimental and sequencing biases, particularly when coverage greatly exceeds what is optimal for assembly. Existing simulated multi-de Bruijn graph assemblers use iterative assembly at multiple kmer sizes to provide a consensus within variable coverage regions. These tools are affected by the quality and level of coverage variability in the data set and often reduce fragmentation while increasing the quantity of erroneous or duplicative contigs that may obscure sequence representing true overlaps.

The quality of *de novo* genome assemblies is limited by the quality and quantity of the input sequences. Without an available reference sequence, the consensus generated by *de novo* assembly can be validated only in the presence of deep and high quality overlaps. Large quantities of sequence data result in greater coverage and more contiguous regions of high confidence overlaps; however, when input sequences contain an extremely high level of redundancy, it may necessitate greater computing resources (e.g., memory, CPU, and disk storage) that are not readily available to many users.

Reducing extremely variable base coverage of reads allows them to be used more efficiently by standard bacterial genome assembly algorithms. The previously published algorithm, diginorm, used RMKF values to predict each read’s coverage, accepting reads until they approach a user-determined cutoff [14]. Additional studies have explored the use of bin separation in coverage-reduced data sets to facilitate memory-restricted assembly of deeply sequenced metagenomic datasets [15]. For implementation of coverage normalization within extremely variable data sets, additional functionality is required to randomize reads targeted for selection, maximize the retention of two-sided mate pairs and give preference to the most unique sequences.

Below, we present a novel algorithm, NeatFreq, for reducing large sequence data sets to uniform coverage, leading to consistently high quality representations of true target sequence using a traditional OLC assembler. The algorithm increases the selection of true, low frequency sequences from a read set which has had maximal false low frequency mers (sequencing errors) removed. For the purpose of this study, kmer normalization is used to separate these false low frequency mers by identifying the prevalence of similar, high abundance mers in the data set [16]. During this process, high frequency coverage peaks are reduced to produce a data set primed for traditional bacterial genome assembly techniques. Reads are binned by a chosen level of retention before selection, after which a user may opt for either random selection or targeted recruitment of the most unique sequences with or without preferential selection of two-sided mate pairs within the set. The pipeline enables the selection of the best available high confidence (quality) consensus sequences from assemblies generated by several OLC single- and simulated multi-kmer de Bruijn graph assemblers while substantially reducing the requirement for high-end computer resources.

## Implementation

### Data

Sequences from the following strains were used in the analysis performed by the pipeline. The format and the type of the sequences were A) Viral samples: 2009 H1N1 Influenza virus single plaque [ftp://ftp.jcvi.org/pub/data/neatfreq_data/HMPVFLU/] and Bacteriophage F_HA0480/Pa1651 [Genbank:SRR407427]- SISPA-optimized titanium 454 fragments, derived from a single plaque [9]; B) Single Cell Amplification of Multiple Bacterial Cells: *Escherichia coli* str. K-12 substr. MG1655 and *Staphylococcus aureus* subsp. Aureus USA300 FPR3757 (reads available at http://bix.ucsd.edu/singlecell/) for which both sequence datasets were obtained by MDA of DNA from single cells that were selectively sequenced from 10 isolates, as chosen by highest exon content. These sequences are therefore expected to be of more normal coverage and greater sequence quality than a true single cell sample [1,2]; C) HMPMDA0100 – Illumina paired-end sequences from a true single cell MDA sample [ftp://ftp.jcvi.org/pub/data/neatfreq_data/HMPMDA0100/]. All samples except HMPMDA0100 have available reference genomes.

### Software dependencies

A pipeline for the configurable auto-curation of all sequence preprocessing stages used in this study is distributed with the open source software package. The recommended pre-processing pipeline includes the third party software cutadapt [17], DUST [18], QUAKE [19], the bio-playground package [20], sffinfo [21], cd-hit-est [22], CLC NGS Cell [23], and ALLPATHS-LG [16]. The Celera gatekeeper program [24] was used for mate-sensitive conversion between file formats with a minimum acceptable sequence length of 40 bp. Tools from the MIRA package were used for fastq file manipulation [25]. Kmer counting was conducted by the program Tallymer from the GenomeTools package [26].

The effect of each preprocessing step on input sequences was captured for all sequencing platform file formats and library types with the assembly quality assessed by rapid assembly using CLC Assembly Cell ver 3.5.5 [23]. The performance of the following assemblers was compared using all reads, pre-processed reads, and multiple levels of coverage reduction: Velvet-SC ver 0.7.62 [5], IDBA-UD ver 1.1.0 [27], and SPAdes ver 2.3 [28,29]. All assemblies were run using sequences converted to either fasta or fastq format. Velvet-SC was run at multiple kmer sizes (k = 25, 35, 45, 55). All other assemblers were used with default settings. Expected coverage values of input sequences required by Velvet-SC were calculated by counting pre-processed query bases aligned to contigs obtained by preliminary CLC assembly using a 40% length and 90% identity cutoff.

### Preparing input files

Read selection using this algorithm requires that input data be cleaned of the most identifiable sequencing errors possible, particularly when using the targeted selection method. Additional screening and removal of contaminants, particularly from samples obtained from human hosts, may be mandated by ethical or funding restrictions. A suggested pipeline, including a preliminary contaminant check, error correction/kmer normalization, exact de-duplication, low complexity/tandem repeat masking, quality trimming, final contaminant check and adapter contaminant removal is described in Additional file 1: Figure S1). Details regarding pre-processing methods used for this study are available in Additional file 2: Supplemental Methods). All datasets in this experiment used 19-mers for RMKF evaluation.

### Reference-free coverage normalization pipeline

The coverage normalization pipeline described here is supplied for standalone use, employing user input kmer frequencies, or as part of a pipeline containing methods for the formatting of all reads and *de novo* calculation of their kmer frequencies. The novel coverage reduction algorithm calculates RMKF values using reports from GenomeTools’ Tallymer [26] and compares them to a RMKF cutoff value provided by the user. Reads with a RMKF = 0, and those with less than K (default = 19) non-ambiguous BPs, are removed from the dataset. RMKF values are calculated for each sequence and compared to a cutoff provided by the user. Each read is placed into one of a count, **Z**, of retention bins (default 100) denoting the percentage of reads to be retained, as follows: 1) All reads with RMKF less than or equal to the cutoff are placed in the 100% retention bin; 2) Reads with RMKF greater than the cutoff are evaluated by the expression, **Retention Bin Selection Value = (RMKF**_**cutoff**_**/RMKF**_**read**_)*Z, rounded up to the nearest integer, and placed in retention bins (1% through 100%) from which the denoted percentage of reads are extracted to satisfy the user-specified coverage cutoff (see the ‘NeatFreq Pipeline Pseudocode’ subsection). Reads with a retention value of less than 1 are rounded up and placed in the 1% retention bin. Reads from the 100% retention bin (least abundant in the original pool) are picked first, followed by progressive selection of reads from the 1% (high abundance) through the 99% (low abundance) bins, utilizing either random or targeted selection of unique sequence.

### NeatFreq Pipeline Pseudocode

- FOR EACH input sequence
  - ○ IF read length, not including ambiguous “N” bps, is greater than kmer size (default K=19)
    - ▪ Update to unique read IDs, Concatenate
    - ▪ Calculate kmer Frequencies
  - ○ ELSE
    - ▪ Delete read
    - ▪ Format broken pairs dataset as fragments
  - ○ ENDIF
- FOR EACH concatenated input sequence
  - ○ (Optional) Build list of mate pair relationships using unique IDs
  - ○ Calculate median kmer frequency
  - ○ Add sequence ID to appropriate “retention bin” by comparing ideal coverage to RMKF
- END FOR EACH
- IF bin selection = random
  - ○ Select all reads from the 100% retention bin
  - ○ FOR each bin (1-99% retention)…
    - Randomize IDs in bin
    - Record IDs up to ideal retention count from bin
  - ○ END
- ELSIF bin selection = targeted
  - ○ Highlight 2-sided mates within bin 1 as high priority
  - ○ Divide all reads by similarity using cd-hit-est
  - ○ Select all reads from the 100% retention bin
  - ○ (Optional) If a mated read is selected, toggle the status of its partner
  - ○ FOREACH retention bin (most selective to least selective)
    - ▪ FOREACH sub-bin cluster (smallest to largest in population)
      - Calculate ideal count of sequences to extract from sub-bin cluster based on count already selected from retention bin
      - IF tracking mates = yes
        - Randomize the sub-bin cluster ID list select from each until the ideal count is reached.
      - ELSIF tracking mates = no
        - Select maximal sets of 2-sided mate pair relationships within sub-bin cluster
        - Select maximal 1-sided mates whose partner has already been selected
        - Select maximal 1-sided mates whose partner has not yet been evaluated
        - Select fragments and 1-sided mates whose partner has been removed
        - Toggle status of all of selected mates
      - ▪ ENDIF
    - ▪ ENDFOR
  - ○ ENDFOR
- ENDIF
- Extract selected IDs from relevant input libraries to maintain fragment and mate pair relationships
- (Optional) Parse 2-sided mates from fragment-only runs using read IDs

### Random selection

**Figure 1:**
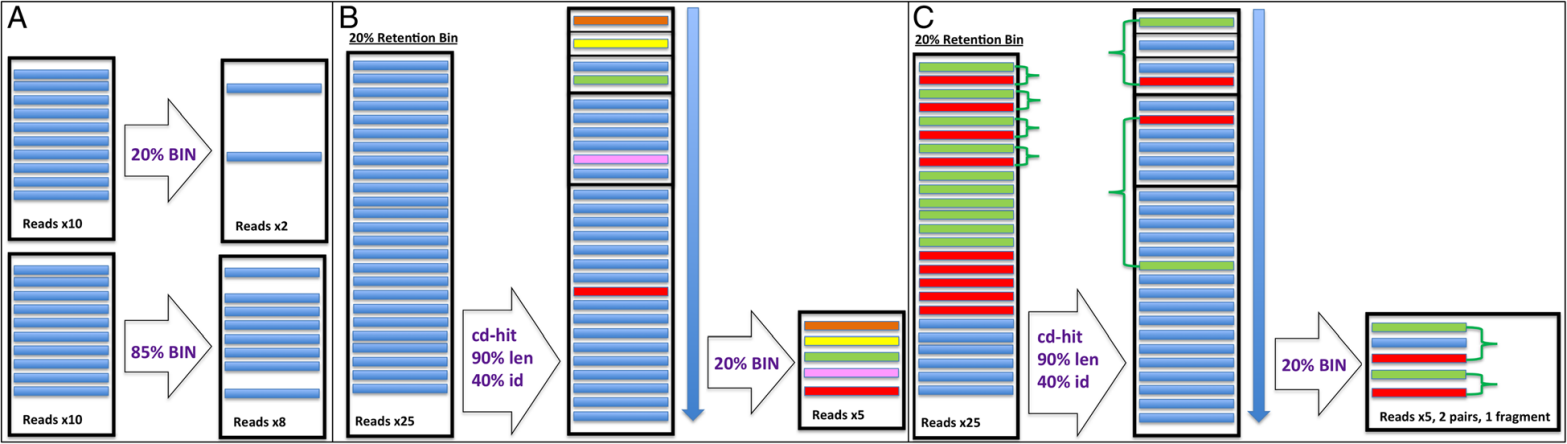
**Schematic illustration of sequence selection by Neatfreq pipeline. A)**. Blue blocks represent fragment-only reads. The left side of the figure shows 2 retention bins (20% and 85% retention) created by the ratio between the RMKF of the read and the cutoff input by the user. The random bin selection method extracts a random subset of reads from each bin up to the count denoted by its retention level and the number of reads available. **B)**. The targeted bin selection method, run as fragment-only sequences, is illustrated on a 20% retention bin (left block). Within each retention bin, reads were clustered by the cd-hit-est alignment [22] program based on similarity and sorted by uniqueness, or the population of the sub-bin cluster (middle block). Illustrated here, reads from each intra-bin sub-cluster were selected randomly from within each cluster approached within a bin. **C)**. Green blocks represent forward mates and red blocks reverse mates, with dark green brackets indicating 2-sided mate relationships. When applying the targeted bin selection method to the same bin containing mate pairs (paired-ends), analysis is identical to that described for fragments except that the retrieval of 2-sided mates across all bins, and their sub-bin clusters, is prioritized. Note that highly unique clusters containing only fragments are still given priority in selection.

The number of reads selected from each bin is determined by the expression: Ideal read count from bin = (Number Of Reads In Bin)*(RetentionBin_**/**_**Total Number of Retention Bins)** (Figure 1A). Sequence IDs are randomized within all bins prior to selection. A random selection of reads from all bins, in inverse proportion to their original abundance, ensures a normalized and uniform selection from all regions of the sequenced genome. If the input sequences contain paired-ends, selected reads can be separated into valid pairs and single-end fragments in the available pipeline, though any two-sided mate retention is purely random and depends on the level of reduction requested.

### Targeted selection

Emphasis is placed on extracting unique sequences from each retention bin to increase the chance of recruiting true low abundant reads which remain after pre-processing. After all sequences from the 100% retention bin are selected, targeted selection begins by clustering reads within each of the remaining bins using the cd-hit-est algorithm (alignment parameters set to 90% identity over 40% sequence length) [22]. Bins are approached one-by-one as in the random bin method and selection from within each bin proceeds iteratively from the most unique sub-bin clusters (smallest population) to the least unique (largest population), as determined by the size of homologous clusters created within each retention bin. Selection from homologous intra-bin clusters occurs by: 1) calculation of ideal read counts for intra-bin clusters with the expression: **(Ideal Read Count per Intra-Bin Cluster) = (Ideal Read Count per Bin, S)/(Remaining Clusters in Bin)**; 2) sorting of intra-bin clusters by size, from most unique to least; 3) randomization of reads within each intra-bin cluster and maximal extraction from each bin up to **S,** and; 4) update of ideal bin and intra-bin cluster extraction counts based on the selected read counts prior to the iterative processing of the subsequent bins (Figure 1B). If the selection was performed without tracking mates for paired-end sequences (i.e. treated as fragments), the mates for the selected reads can be parsed after reduction as described for the random method above.

When input includes paired-end reads and the targeted bin selection method is chosen, reads representing one side of a mate pair relationships are tracked by their selection status and the status of their mate. Two-sided mate pair relationships found within a single bin or intra-bin cluster are given the highest priority during selection, followed by mated reads with unresolved one-sided relationships. During the initial recruitment of all reads in the 100% retention bin, one-sided mates are toggled to indicate the possible recruitment of both pairs in the two-sided mate relationship. Subsequently, as selection within the 1% (to 99%) bin proceeds, new one-sided mates are evaluated by their corresponding mate status and are preferred for selection if their mate has previously been selected (Figure 1C). One-sided mates whose pair has already been discarded are reduced to the priority of fragments.

### Assembly statistics

All assembly statistics evaluate contigs greater than 500 bp only to comply with NCBI assembly submission requirements. Assembly coverage statistics were calculated. The successful retention of low coverage sequence in following dataset reduction is evaluated using the change in represented reference bases when aligned to the shredded consensus sequence (Columns ƍ, §, ¥: Additional file 3: Tables S1, S2). Average contig coverage was calculated as a weighted mean of means across all contigs based on contig length. Changes in the sequencing span (reference bp in sequences) and assembly span (reference bp in contigs) at each pre-processing stage were calculated by aligning either query sequences or the assembled output from each stage of pre-processing to the available reference. Contig and sequence alignments were evaluated by aligning to the reference at 40% length and 90% identity cutoffs with contigs shredded to 7999 bp with 49 bp overlaps. Compute time was captured using system time on a single host machine with 4 CPU and 256 GB RAM. Memory usage was monitored by ‘ps’ with runs executed in isolation on a single host.

## Results

Short-read assembly requires deep coverage to in order to sufficiently sample the source genome since shotgun sequencing is subject to random sampling variation, amplification and systematic sequencing biases. Some of the recently developed random-primed laboratory techniques like MDA and SISPA enable whole genome sequencing of organisms that cannot be cultured, but have the caveat of highly variable sequence coverage due to amplification biases [9,30]. As described by Brown et al. [14], the relationship between the RMKF of each read and its true coverage can be estimated with a 1% error rate on simulated sequences. Production of the longest valid consensus span requires sufficient coverage across the entire genome; however, due to selection, amplification and sequencing biases, it is likely that certain regions yield far more coverage than others, especially with extremely deep sequencing coverage (e.g., above 400-fold).

### Prioritized selection of true low coverage sequences

**Figure 2:**
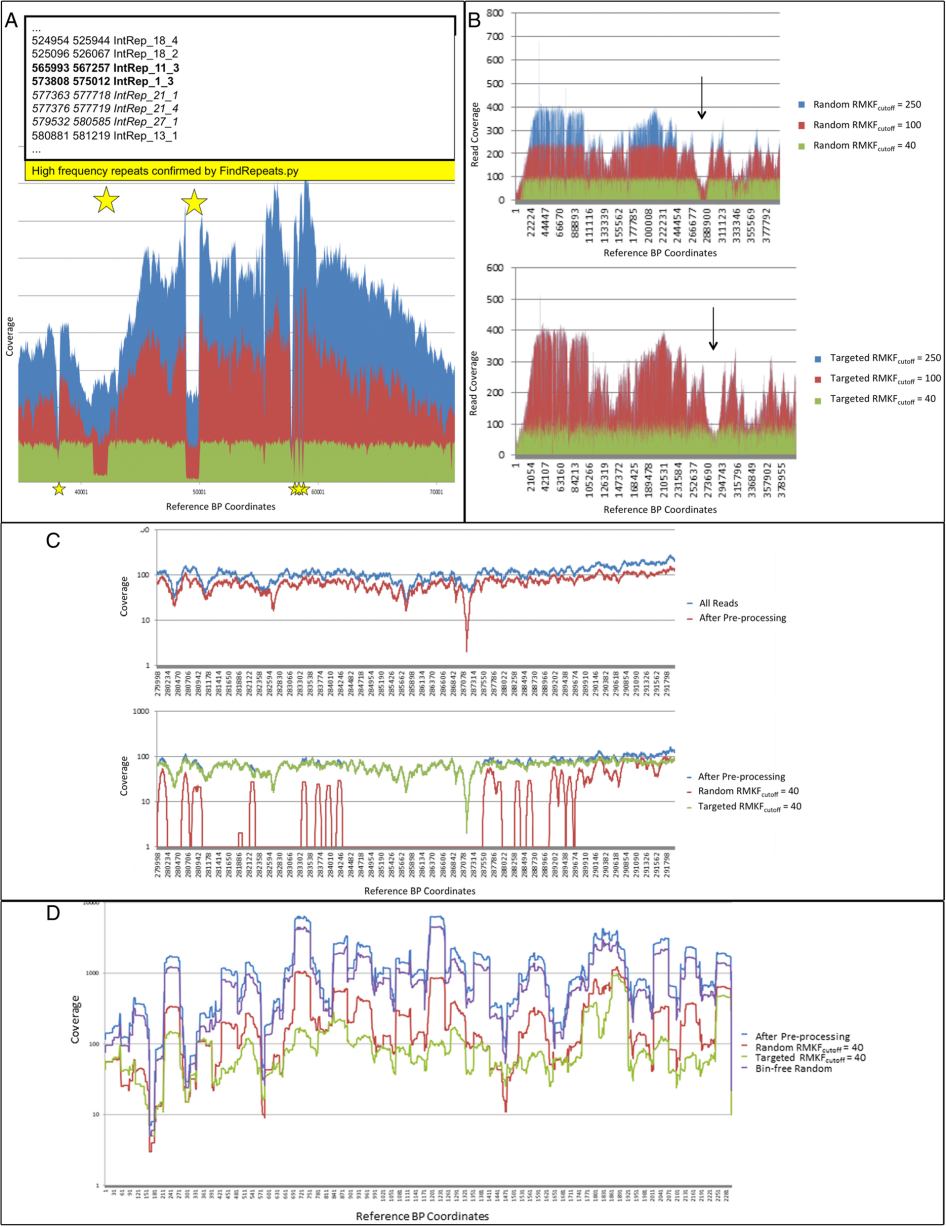
**Comparison of random and targeted NeatFreq selection methods on sequence coverage. A)**. Elevated kmer counts within repetitive regions cause over-reduction using an RMKF cutoff. The genomic regions labeled with stars indicate regions identified as repeats by RepeatFinder [30]. Reads from repetitive regions are placed in low selectivity bins due to the high frequency of similar mers within the data set. Therefore, over-reduction occurs at multiples directly related to the count of repetitive regions. **B)**. This histogram shows the retrieval of sequences at different RMKF cutoff levels when using each of the bin selection methods. Aligned sequence coverage distribution is shown for the first 40,000 bp of the *S. aureus* genome using query sequences selected by random (top) and targeted (bottom) methods. The targeted method is more effective at recruiting low coverage regions resulting from single cell amplification bias in variable coverage region, including 0-fold regions. The X-axis shows genomic coordinate from the reference used for mapping the extracted reads and the Y-axis shows the level of coverage at each genomic position. **C)**. The histogram gives zoomed view of the low coverage area highlighted by an arrow in Figure 2A (region 278 kbp – 292 kbp). Alignment histograms show that the targeted algorithm, in contrast to the random selection, retains the low coverage areas in the variable dataset, resulting in an increased sequencing span. **D)**. Coverage histogram of reads aligned to the largest H1N1 Influenza genomic reference segment (log scale). Random selection from the entire dataset (without retention bins) was performed to a count of reads equal to that used by targeted selection at RMKF cutoff =40. This random selection from all reads is the most subject to input coverage variability and fails to reduce deep spikes to generate coverage levels compatible with OLC assembly.

The most common reasons to normalize read coverage are to: eliminate duplicate reads, minimize sequence errors, recruit more reads within low coverage regions and minimize computational and memory resources required for assembly. Traditional OLC bacterial assemblers like Newbler and Celera WGS prefer 40-80-fold of uniform coverage across a single genome. Both algorithms often fail during consensus resolution of genomic regions with particularly high coverage peaks, as shown by the failed experimental single cell sample assemblies missing in Additional file 3: Table S2. The reduction to normalized sequence coverage was shown to promote completed assembly of the experimental HMPMDA0100 sample in contrast to the failed assembly when more sequence data was used (failed cases not shown in table). The degree to which reads from each of the retention bins are represented in the final pool varies with the coverage complexity of the dataset and the RMKF_cutoff_ value supplied by the user. Kmer frequencies are not merged for forward and reverse compliments, so RMKF_cutoff_ correlates empirically to approximately half of the expected output sequence coverage when using the random bin. As each retention bin can contain reads with varied abundance, the likelihood that less abundant sequences are not selected is high when using the random bin selection method, particularly with extremely variable coverage input sequences. Both random and targeted bin selection methods discriminate against the selection of repetitive sequence because their representative kmers are overabundant in the overall data set and recruited at a lower priority. As illustrated in Figure 2A, reads were found to be recruited at less than normal coverage within regions of the reference identified as repeats using RepeatFinder [30].

Changes in assembly and sequencing spans showed that the pre-processing steps caused the loss of some reference bases, particularly when aggressively removing erroneous reads by read correction (Columns §, ¥: Additional file 3: Tables S1, S2). Despite lost reference bases in reads, preliminary read processing to remove false low frequency mers was shown to improve the inclusion of low coverage regions in the final assembly, particularly when handling datasets with many low coverage regions or with a wide range of coverage across the genome span. Using all pre-processed sequences as input, reduction to assembly-ready coverage removed as much as 90% of the reads for samples found to have excessive coverage (Additional file 1: Figure S2A), while minimally removing true reference bases within low coverage regions (Columns §, ¥: Additional file 3: Tables S1, S2).

### Improved selection for low coverage MDA sequence or scaffolding

Random bin selection used less memory and was significantly faster than the targeted alternative, making it an ideal option for large prokaryotic genome assemblies with only moderate coverage variability. For larger datasets, Newbler assemblies used less system time and RAM for all assembly and processing after data reduction was performed by the recommended preprocessing pipeline or following coverage normalization (Additional file 1: Figure S3). The effects of random and targeted bin selection algorithms on the coverage reduction and normalization of the over-sequenced H1N1 Influenza viral plaque sample were compared to all unprocessed reads, pre-processed sequences, and a completely random subset of the pre-processed data set. Targeted selection from bins minimized the loss of true low coverage sequences when evaluating samples with pronounced coverage differences (Figure 2B). The likelihood that some low abundant sequences are not selected by random bin selection was linked to sequencing coverage, and true reference base-pairs may be removed within low coverage regions, inhibiting the extension of an assembled consensus through that region of the genome (Figure 2C). Targeted selection from retention bins by homologous clustering increased the likelihood that reads coming from low coverage regions were represented in the selected output pool, resulting in fewer missing reference bases compared to random selection methods. In this context, a completely random selection of the reads failed to remove problematic coverage spikes, while the targeted selection (sampling) from homologous sequence clusters retained more reads from low coverage regions, resulting in an improved genome span as evaluated by comparison to a reference genome (Column ¥: Additional file 3: Table S2). Targeted reduction displayed the best approximation to true genome span for all samples requiring coverage reduction by recruiting more reads to low coverage regions (Column Ħ: Additional file 3: Tables S1, S2). A comparison of bin-based selection for reduced coverage to bin-less random selection from all reads was performed, showing a tendency of the latter process to follow the coverage distribution of the input dataset and a failure to remove coverage spikes, which inhibit OLC assembly (Figure 2D).

Targeted selection using mate tracking recruited 10-20% more two-sided pairs than when reads were initially selected from bins as fragments and followed by the extraction of their mates (Additional file 1: Figure S2B). The prioritized selection of mate pairs was shown to be successful at creating improved scaffolds, linking fewer contigs (Columns α, β: Additional file 3: Table S3). This effect was more dramatic in the OLC assemblies than those generated by the scaffolded multi-de Bruijn graph equivalent. Here, scaffolding of both control MDA samples (*E. coli*, *S. aureus*) was improved despite losing as much as 45% of the two-sided mate information.

### Validation of coverage normalization pipeline on controls

**Figure 3:**
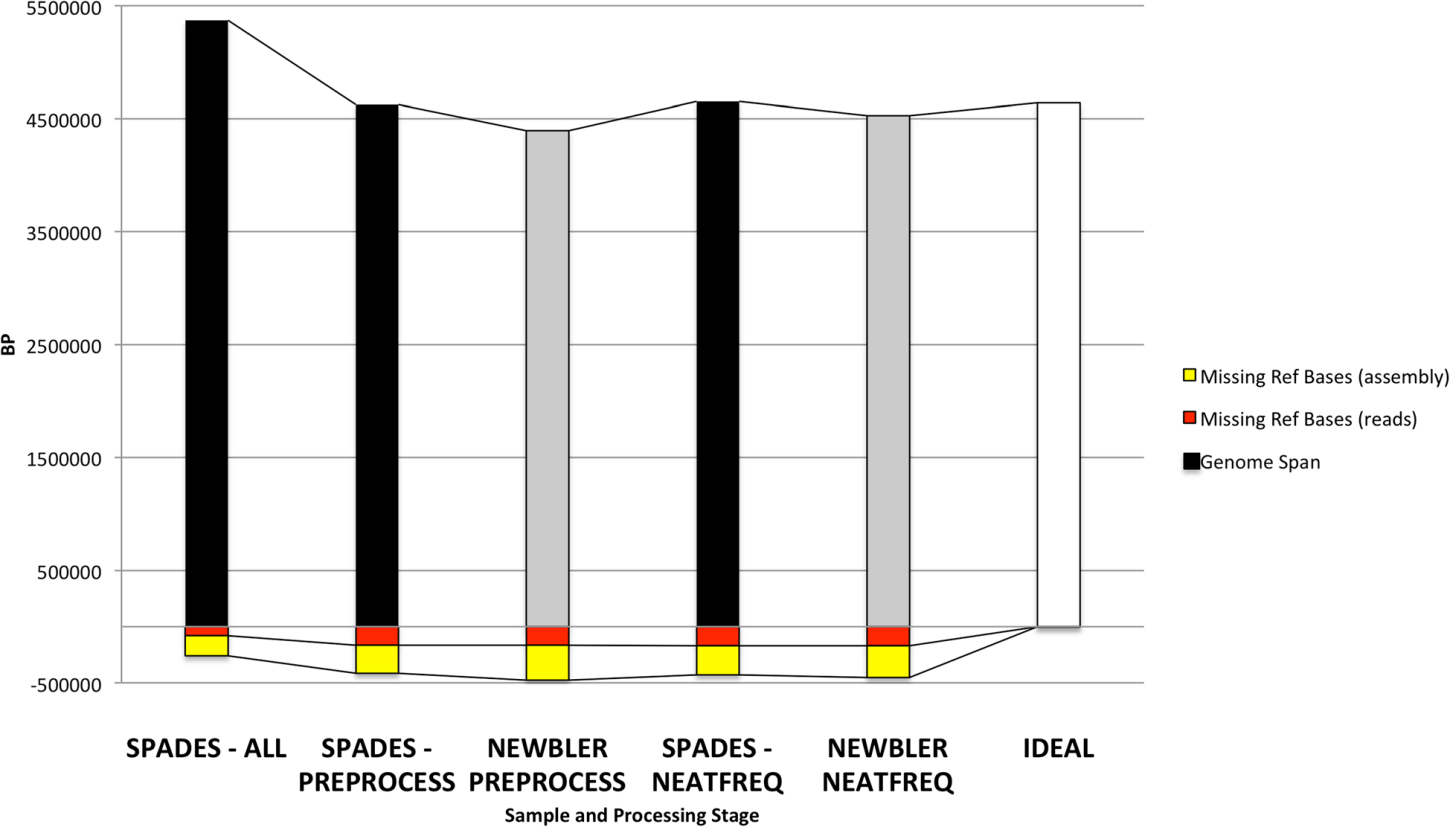
**Comparison of** ***E. coli*** **MDA assemblies to the expected (ideal) genomic span.** Newbler assembly with all reads (ALL) fails on all samples during consensus resolution (not shown). SPAdes is able to complete an assembly using all reads with few missing reference bases in the assembly but the output also generates excessive redundant and spurious contigs. Pre-processing reduces sequences allowing the completion of OLC (Newbler) assembly and yields genomic spans by both Newbler and SPAdes which are most similar to the ideal (reference) assembly. The Newbler assembly from coverage-reduced data is also closer to the reference span and verifies (consistent with) the assembly produced by the SPAdes following preprocessing alone.

Samples amplified by MDA and built from multiple cells (*E. coli*, *S. aureus*) saw the largest jump in the loss of base coverage (vis-à-vis reference bases) using AllPaths read correction, although the resulting CLC assembly of these reads aligned to the reference sequences at levels comparable to SPAdes assembly of all reads (Columns §, ¥: Additional file 3: Table S1). Samples requiring additional coverage reduction, specifically *E. coli* as compared to *S. aureus*, assembled into a more contiguous consensus at closer approximation to the reference genome span. Similarly, for the experimental single cell sample, HMPMDA0100, in which the entire genome was not fully represented, average coverage decreased as low quality bases were trimmed or corrected out of the assembled data pool and highly abundant spurious contigs were removed from the assembled output (Column Π, Additional file 3: Table S2). A comparison of bin selection methods for sequence selection showed an increased number of formerly low coverage regions receiving at least 5-fold coverage following reduction with the targeted method across all samples except HMPMDA0100 (Column ƍ: Additional file 3: Tables S1, S2). In this case, random reduction collapsed the overlap graph and produced a consensus at less than half the predicted size, resulting in inflated coverage values. SPAdes assembly of the deeply sequenced control MDA *E. coli* sample yielded the most reference bases when using all reads, but did so at the cost of generating approximately 730 kbp of nearly duplicative contigs containing errors (Figure 3), In these cases, SPAdes output consensus lengths were found at 108% and 1048% of expected genome spans for *E. coli* and bacteriophage F_HA0480/Pa1651 samples, respectively (Column Ħ: Additional file 3: Tables S1, S2).

For the bacteriophage and influenza samples with a short genome span, as well as the idealized high quality MDA *E. coli* sample, the cumulative metrics contig N50, consensus span, difference from reference genome span, extent of missing reference bases in contigs and the percent of contig duplication suggest that sequences selected by both random and targeted methods yield assemblies that are improved or highly similar in quality to the assembly of all reads (Additional file 3: Tables S1, S2). Moreover, these assemblies yielded consensus spans at closer to the reference genome size while increasing the inclusion of reads from lower coverage regions, specifically those found at a minimum of 5-fold coverage, and reduced the fragmentation of the output consensus (Column ƍ: Additional file 3: Tables S1, S2). As coverage is calculated by the alignment of the input reads to the assembled consensus, average coverage was shown to increase when low quality and contaminant sequences were removed from the datasets. Changes in the sequencing (**§**) and assembly span (¥) for individual samples showed that despite removing true reference bases in reads during NeatFreq reduction compared to the pre-processed input, the resulting assemblies produced an increased (influenza, *E. coli*, *S. aureus*) or competitive (bacteriophage) assembly span (Additional file 3: Tables S1, S2). When deep coverage differences were normalized by random or targeted coverage reduction, Newbler generated assemblies of comparable quality with less duplication and fewer spurious contigs relative to SPAdes and other simulated multi-de Bruijn assemblers from all samples.

The successfully executed Newbler assemblies also showed a comparable count of 0-fold (missing) reference bases as observed from the best-simulated multi-de Bruijn results for highly variable inputs. Unlike SPAdes and Velvet-SC, the Newbler assemblies either failed to run to completion when all unprocessed reads were used for high coverage samples (*E. coli*, and *S. aureus* and coverage-variant HMPMDA0100) or produced lower quality results prior to the data reduction and coverage normalization (Additional file 3: Tables S1 & S2). SPAdes used lower peak memory and less processing time than is required for the suggested pre-processing-NeatFreq-Newbler pipeline for the bacteriophage sample, even when all reads were used as input (Additional file 1: Figures S3A, S3B).

## Discussion

The novel algorithm described here adapts and extends the earlier methods of digital normalization using kmer frequency analysis by (1) selecting reads randomly within retention bins using cutoffs set by expected levels of reduction, (2) providing the optional selection of the most unique sequences in a dataset by comparing the contents of scaled retention bins and (3) offering optional extraction of two-sided mate pairs during the reduction of sequences to obtain normalized coverage. Effective pre-processing reduces false kmers in the dataset and allows the removal of exact duplicates while minimizing memory use and the obfuscation of alignments due to the presence of superfluous sequences. The reduction of overabundant sequences and spikes in unequally distributed coverage across a target genome were found to build improved consensus sequences when assembled by both OLC and simulated multi-de Bruijn graph algorithms. The targeted bin selection approach offers a novel method for the recovery of rarely occurring sequences critical to samples with extreme coverage variation such as those generated from the sequencing of randomly amplified genetic material.

When large data sets inhibit processing due to algorithmic or resource restrictions, the ideal starting point in reducing coverage is the removal of sequencing errors. The exclusion of erroneous kmers results in the improvement of assemblies when samples are over-sequenced, but exhibit no significant variation in coverage (Additional file 3: Table S1 – *S. aureus*). The goal in each *de novo* assembly task must be to develop an effective strategy to remove uninformative sequences in a way in which the biases inherent to individual sample are best addressed.

The choice between random and targeted bin selection methods for normalization of extremely variable sequence data is dictated by the nature of the sample. The random bin selection method is preferable to taking completely random subsets of data in all cases where deep coverage spikes prevent successful assembly (Figure 2D). This selection method uses less memory and runs more quickly since it neither tracks mate pair relations nor requires intra-bin clustering by similarity (Additional file 1: Figure S3B). As such, the random bin selection algorithm is preferable to the targeted method for common sequencing analyses which may utilize deep sequencing, but do not require specialized retention of low coverage regions. By nature of the randomized selection within each bin, the highest population reads within each predicted coverage level are also more likely to be retained in the extracted sequence set when using this selection method. For this reason, the targeted selection method should be preferred for the recruitment of these low frequency kmers for the assembly of extremely variable coverage sequence, such as that found in transcriptomic data sets or prokaryotic sequence exhibiting MDA or SISPA bias. Additionally, the targeted bin selection method may be preferred for its capability of preferential 2-sided mate pair selection at the cost of time- and resource-intensive processing. Such targeted selection should be used only with those samples that have been error-corrected by preliminary kmer normalization. This allows for the effective removal of exact duplicates without increasing the output of short, erroneous, or chimeric kmers (Additional file 1: Figure S4), and increases the predicted coverage for valid kmers in the kmer graph. As currently implemented, the RMKF-based coverage prediction approach is primarily intended for analyzing data obtained from individual species. Should multiple species be present in a data set presented to the NeatFreq algorithm, each would reduce to normalized coverage, thus increasing the relative population of any low abundant organisms in the reduced output. In addition, the presence of sequences from multiple species introduces noise in kmer frequency counts, which may result in reduced coverage in the target organism. Following reduction, the relative increase in coverage for low abundant contaminating sequence, as compared to target sequence, further facilitates greater contiguity in contaminant assembly for improved identification, recursive removal and reduction. Furthermore, the use of these kmer frequency bin selection reduction algorithms with mixed sample data (e.g. metagenomic) requires a preliminary binning to separate organisms by taxonomy, GC profiles, etc. for optimal use. These techniques, however, would not be sufficient for the separation of similar strains where normalization of similar kmer profiles would complicate true overlaps within organisms present at different coverage levels.

The large amount of data generated from multiple samples by high throughput sequencing methods necessitates an automated, yet flexible processing pipeline. Ideally, the pipeline should perform recursive analysis using several methods of read selection and assembly algorithms, allowing one to choose from the most improved genomic consensus sequences. By tracking the input sequence metadata and the subsequent assembly results, a user or program can quickly evaluate the effects of specific pre-processing steps on the assembly quality, particularly when a reference is available. Furthermore, automation of sequence pre-processing and coverage reduction allows rapid processing of samples while conforming to reduced computer memory and data storage resources available to most users.

The pipeline described here has a number of other uses, including the preparation of Illumina sequences for the correction of lower quality, longer read length sequencing types including those from PacBio to ensure fewer erroneous bases in the final assembly. The tool can also be useful in the *de novo* assembly of transcriptome sequences including those of low abundance isoforms with the caveat that no differential expression analysis is possible due to the inherent normalization of sequence coverage. However, in the future, an implementation of extended read tracking could allow for automated *de novo* gene finding and expression analysis in RNA-seq projects. As currently implemented, the fragment-only pipeline can be used to process sequences and genomes of all sizes. Processing using paired-end reads should be capped at 100 million pairs due to lack of parallelization in the execution of some stages in the pipeline. The planned improvements incorporating multi-threading and multi-processor (CPU) options and the parallel processing of bin calculations in a cluster environment (SGE, Cloud) would extend its utility in processing larger datasets, including higher eukaryotic samples. Additional improvements could be made to compare the assemblies produced by the pipeline to known insertion sequence elements in order to detect and resolve known issues of sub-normal recruitment of repetitive sequences. The software can be trained with the expected kmer distributions from multiple related reference sequences for metagenomic data or a single reference for individual novel samples. Implementation of the algorithms and pipeline as described is valuable in delivering high quality assemblies from high-density data, obtained from prokaryotic and small eukaryotic species containing extremely deep coverage differences.

## Conclusions

The single cell amplification of novel organisms whose genome span is unknown and contains sequences of extremely variable coverage requires approaches that emphasize data reduction and coverage normalization prior to their use with high-confidence OLC assemblers such as Newbler and Celera WGS to generate more valid assemblies of target genomes. In this study, we have shown that using OLC methods with a reduced set of high quality sequences results in conservative assemblies that can be used as a standard to validate results obtained from more aggressive assembly programs that require all reads as input. Simulated multi-de Bruijn graph-based assemblies using multiple kmer sizes such as SPAdes, Velvet-SC and IDBA-UD perform well with samples of deep coverage using input sequences that have not been pre-processed. When using all input reads and comparing to the same assemblies using reads reduced by either the random or targeted selection methods, these simulated multi-de Bruijn graph assemblers, which expect variable coverage input sequence, are shown to output assemblies with more consistent confidence when compared to the reference genome. Pre-processing of input sequence reduced assembly resources and generated assemblies that were less fragmented and contained fewer spurious contigs (Column Ħ: Additional file 3: Tables S1, S2; Figure 3). Verification of the sequencing spans generated by these simulated multi-de Bruijn graph assemblers by the concomitant high confidence OLC assemblies allowed for the selection of the most valid consensus sequences and minimized time and effort spent for the costly post-assembly analysis (finishing) to eliminate contigs of dubious quality. Our analysis also demonstrates the utility of extracting mate pairs (or paired-ends), when available, and finds that the selection of unique sequences over a wide range of coverage depth may allow for more contiguous assemblies with improved scaffolds using commonly problematic data sets.

## Availability and requirements

**Project name:** NeatFreq

**Project home page:**https://github.com/bioh4x/NeatFreq

**Operating system(s):** Unix/Linux

**Programming language:** Perl

**Other requirements:** 3^rd^ Party Utilities (Install Instuctions Provided)

**License:** GNU GPL 2

**Any restrictions to use by non-academics:** None

## Additional files

Additional file 1:
**4 Supplemental figures described in text.**
Additional file 2:
**Supplemental Methods.** A summary of supplemental methods for NeatFreq preprocessing. Additional file 3:
**Supplemental tables of assembly and scaffold statistics described in text.**
