# Supplementary material for: NeatFreq: reference-free data reduction and coverage normalization for De Novo sequence assembly
Source: BMC Bioinformatics. 2014 Nov 19;15(1):357. doi: 10.1186/s12859-014-0357-3 (PMC4245761; doi:10.1186/s12859-014-0357-3)
Supplement: Additional file 1: — 4 Supplemental figures described in text. [file 12859_2014_357_MOESM1_ESM.pdf]

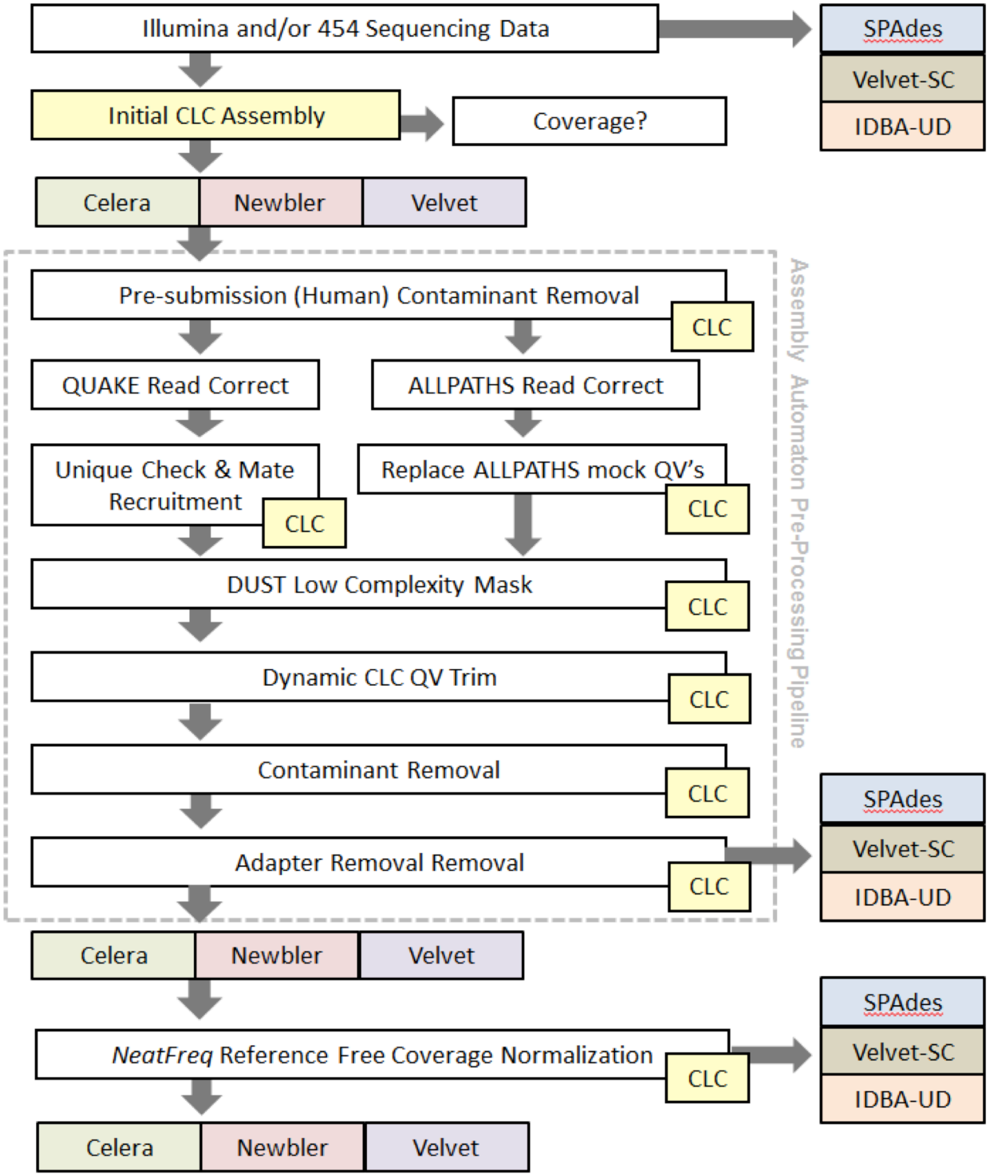

Supplemental Figure 1 : Automaton Preprocessing Pipeline

The "assembly automaton" processing pipeline, enclosed in the dashed box, contains a series of optional analysis stages (shown in solid rectangle boxes) which can be executed as an automated pipeline. The sequences obtained at each stage are assembled by the CLC *de novo* assembler to track and monitor the quality of assemblies as judged by the total contig span. During this analysis, one or more of the simulated multi-kmer de Bruijn assemblers (SPAdes, Velvet-SC and IBDA-UD) and conventional assemblers (Newbler, Celera WGA and Velvet) are used before and after preprocessing and coverage normalization to evaluate changes in assembly quality.

A.

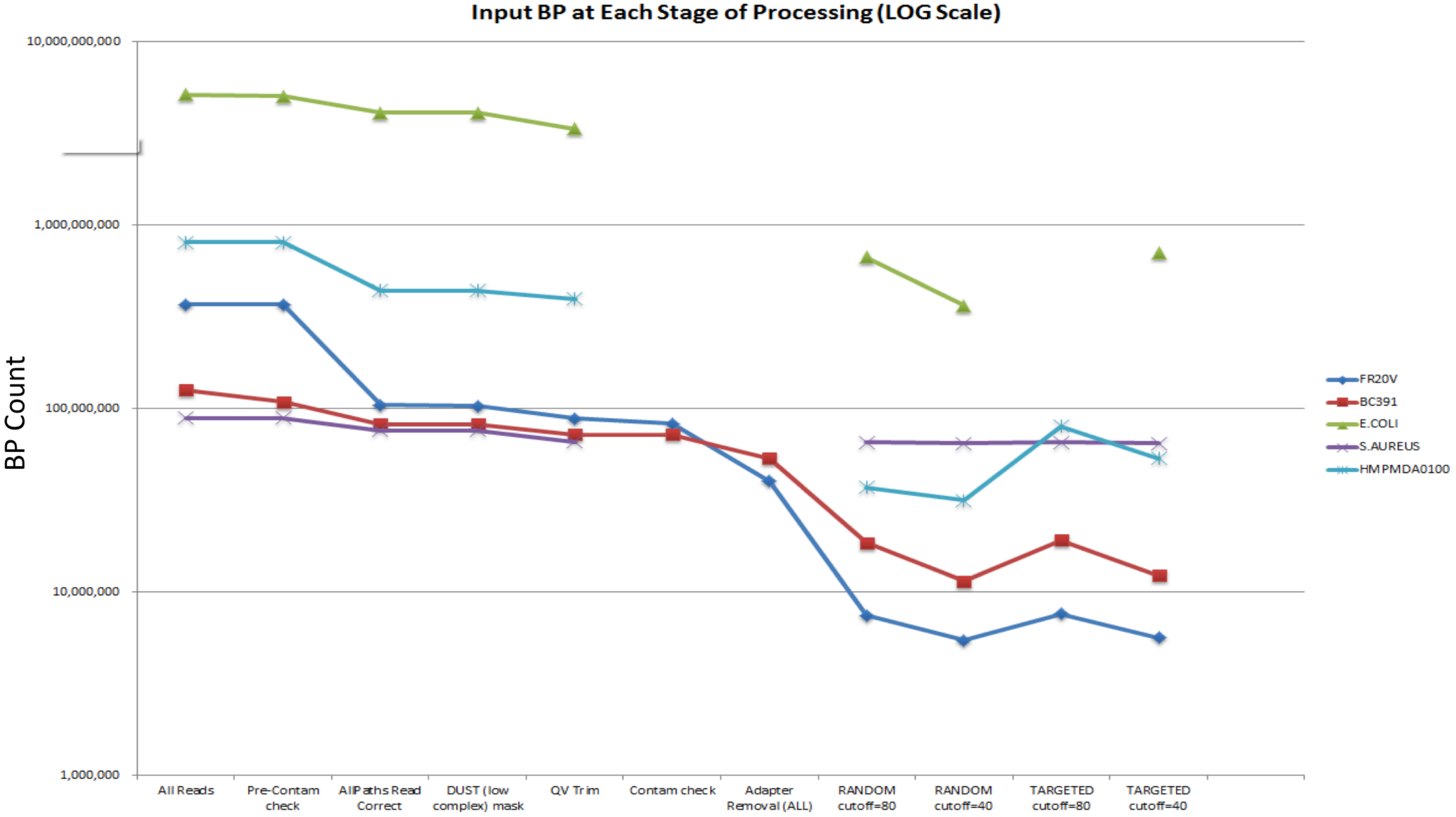

B.

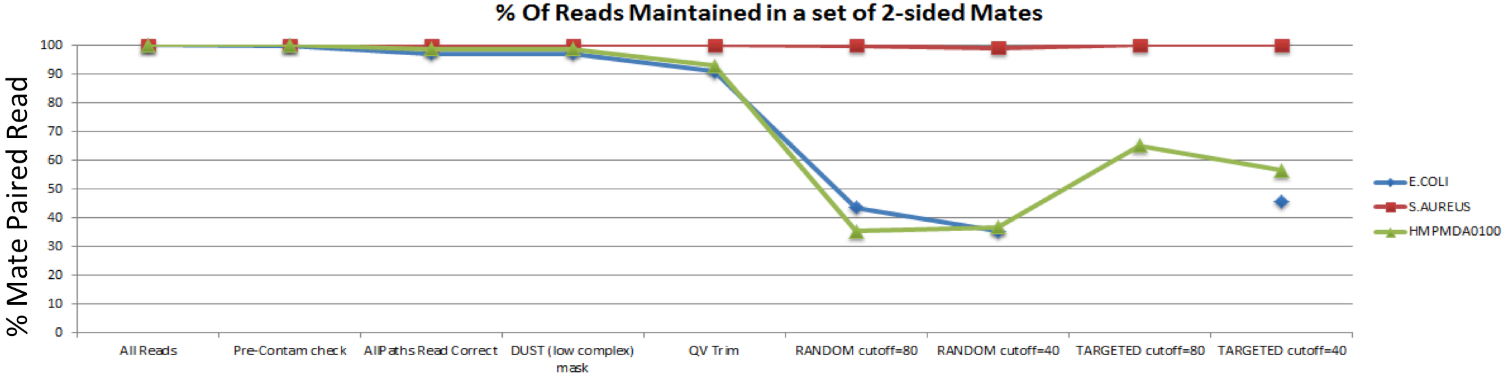

Supplemental Figure 2 : Read Retention During Processing

A). Input counts of base pairs in all sequences used in assembly are shown, in LOG scale, for each stage of preprocessing used in experimentation across all samples. Reduction occurs at levels relative to the target genome span.

B). The percentage of reads which represent one or both parts of a valid, 2-sided mate pair relationship are shown for each stage of processing used in experimentation. Increased 2-sided mate retention coincides with improved scaffolding in **Table 3**.

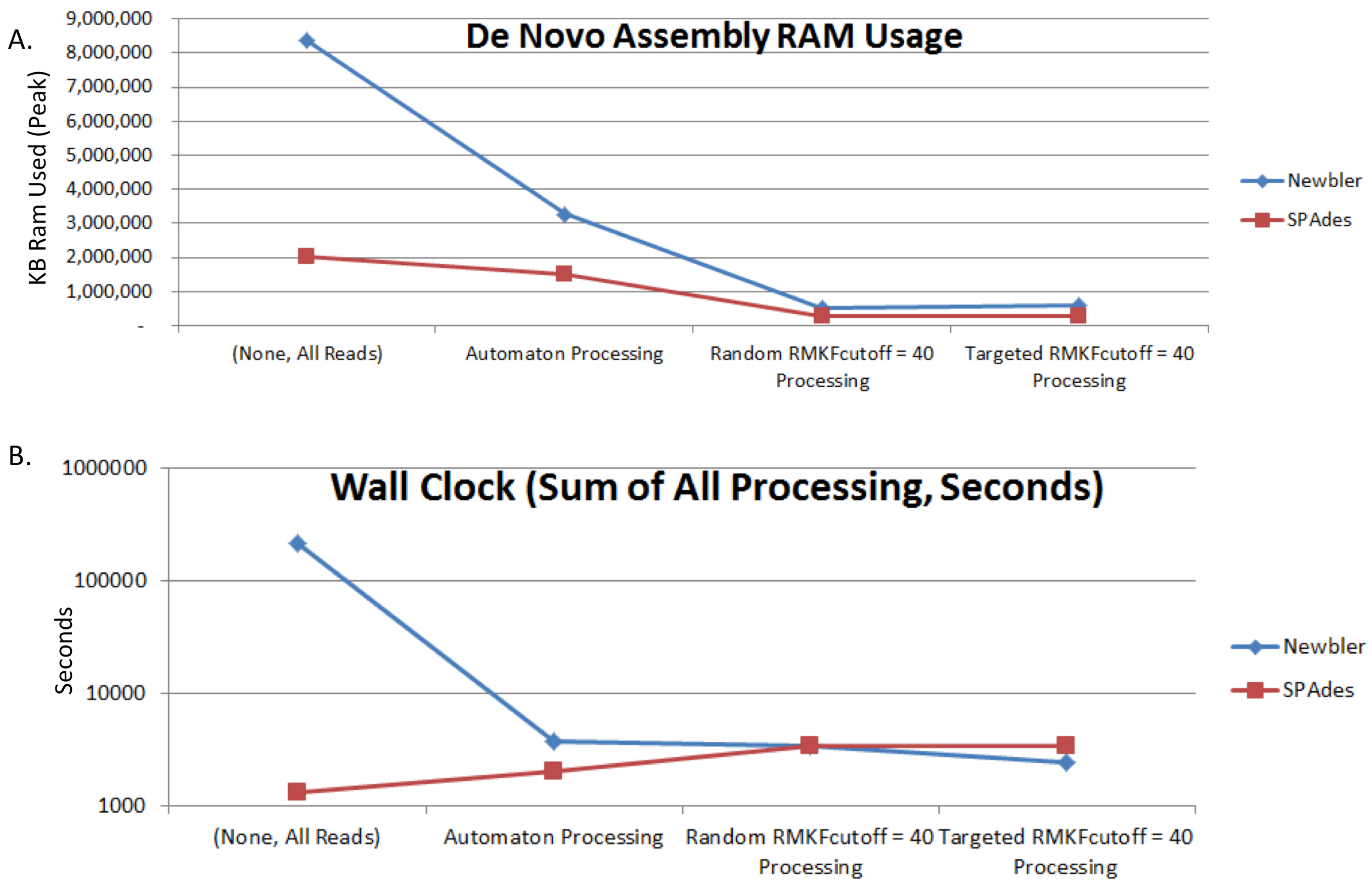

Supplemental Figure 3 : Resource Utilization during Processing

A). Peak RAM usage during all processing steps, including assembly with Newbler and SPAdes algorithms, are shown.

B). The total time used by all processes (pre-processing and assembly) for bacteriophage F_HA0480sp/Pa1651 is shown.

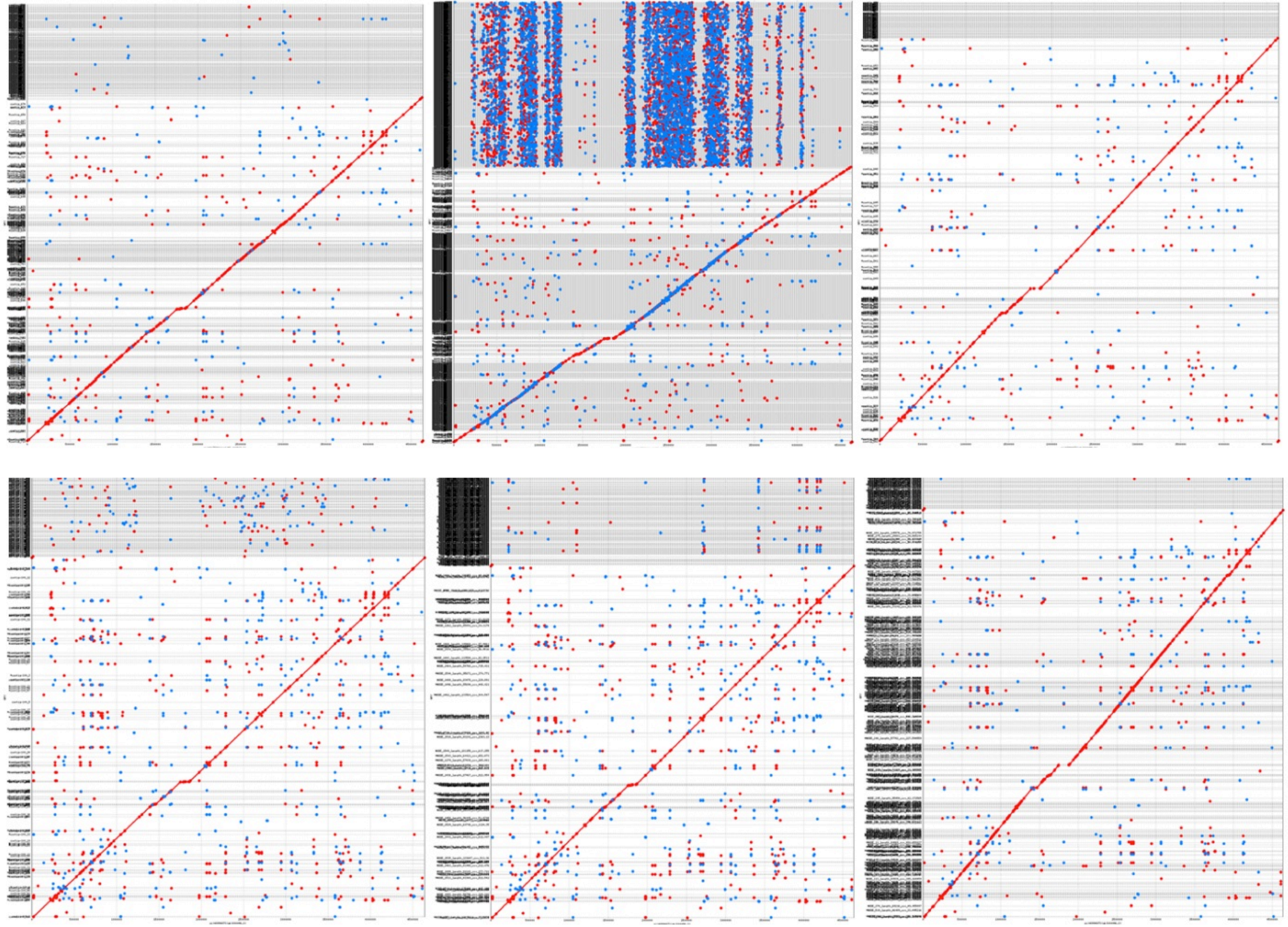

Supplemental Figure 4 : Chaff/Duplicative Contig Outputs Before/After Deduplication
Alignments were curated by the mummer package with alignments rendered using mummerplot [21] . The Y-axis represents input contigs from control MDA processing and the X-axis represents the *E. coli* reference genome. On the **top row (left to right) :** Contigs represent CLC assembly of (1) all reads, (2) all reads following exact de-duplication, (3) all reads following AllPaths read correction and exact de-duplication. The removal of exact duplicates before correcting bad kmers causes the production of duplicative chaff contigs. On the **bottom row (left to right)**. Contigs represent assembly of all reads using (1) IDBA-UD, (2) SPAdes, and (3) Velvet-SC K=55. Assemblers using multiple kmers improved approximation to the correct genome consensus while avoiding the de-duplication error compared to the CLC all reads equivalent, but not compared to a CLC equivalent that had been read-corrected.
