# Supplementary material for: NeatFreq: reference-free data reduction and coverage normalization for De Novo sequence assembly
Source: BMC Bioinformatics. 2014 Nov 19;15(1):357. doi: 10.1186/s12859-014-0357-3 (PMC4245761; doi:10.1186/s12859-014-0357-3)
Supplement: Additional file 2: — Supplemental Methods. A summary of supplemental methods for NeatFreq preprocessing. [file 12859_2014_357_MOESM2_ESM.docx]

**Supplemental Methods :
Preprocessing Pipeline (Assembly Automaton)**

The distributable package includes a Perl wrapper script that automates and tracks the execution of third party software packages for “push-button” preprocessing and the assembly of sequences. Users are given the option of toggling each stage of preprocessing on or off and provided output to monitor the effect of such actions on the input sequences and the quality of subsequent assemblies (**Supp. Figure 1, Supp. Table 1**). The tool can be executed on a single UNIX host and requires that all third party tools be installed and configured separately. The pipeline accepts SFF or Illumina FASTQ formatted sequences either as fragment or paired-end sequences (innies), each restricted to one insert size (library) per each sequence format. Additional libraries can be accommodated by tracking read IDs. Libraries comprised of mate pair sequences (outties) may be reverse-complimented before and after processing to satisfy the paired-end only requirement prior to their use in the pipeline. The various components of the pipeline are executed sequentially with harcoded options set as follows: **A) Contamination check I** – an optional first step performed to remove sequences that align to the user-provided contaminant reference fasta sequence database with 95% identity over at least 40% of the query length. Alignments are performed by the CLC reference assembler following the conversion of SFF and FASTQ sequences to FASTA format with sffinfo [20] and the utilities available in MIRA assembler package [24], respectively. One-sided mates resulting from any of the preprocessing steps are retained as fragments for all downstream analyses; **B) Error Correction** (using Illumina sequences only) – a mandatory step of user-selected QUAKE [18] or Allpaths-LG [15] kmer-based read correction algorithms. If the data includes both 454 and Illumina sequences, the kmers from the former are not considered during the error correction of the Illumina data. Fragment-only Illumina sequences are provided to the Allpaths-LG error-correction module as mock mates reduced to an even count, since the program can only accept paired-ends, and reverted to single-end status following the analysis; **C) Deduplication** – an optional step for Illumina sequences only. The removal of the exact duplicates by the *fastq* package [19] is performed separately for fragment and paired reads, with the latter separated into fragments (orphaned pairs) and the surviving mates following the analysis; **D) Low complexity masking** - an optional step performed on both SFF and FASTQ sequences using the DUST package [17]. As DUST utilizes fasta sequences only, quality scores from the original fastq or the converted SFF equivalent are retained and re-applied to the masked output bases; **E) Quality trimming** – a mandatory process on all Illumina sequences. The dynamic quality trimming is performed using CLC NGS Cell’s [22] quality_trim tool with a quality score cutoff of 18 and a cutoff of 2 contiguous N base calls; **F) Contamination check II** – an optional procedure performed identically as described in the first step – removes previously undetected contaminant sequences that can align to the reference sequences only following the error correction and quality trimming stages, and finally; **G) Adapter** **removal** – an optional step performed on both SFF and FASTQ sequences using cutadapt [16] in three serial stages for each provided contaminating adaptor sequence. These steps are set up to allow for aggressive removal of partial SISPA barcodes represented by as few as 1 bp or as many as 20 bp and thus result in a reduction of average read lengths for all libraries considered [9]. The forward and reverse-compliment of the adaptor sequences are aligned to the 3’ and 5’-ends of the input reads and matches up to a minimum of 6 bp with 100% identity are trimmed. Finally, the reads with near full-length adaptor matches found in the middle of the sequences, representing potential chimeric joins, are removed from the dataset. After each preprocessing step, the sequences are assembled using CLC assembly cell version 3.5.5 [22] and various statistics are calculated on the resulting contigs to track positive and negative changes in the output and provide values for settings, such as expected coverage and genome span for automated assembly.
