# Supplementary material for: NeatFreq: reference-free data reduction and coverage normalization for De Novo sequence assembly
Source: BMC Bioinformatics. 2014 Nov 19;15(1):357. doi: 10.1186/s12859-014-0357-3 (PMC4245761; doi:10.1186/s12859-014-0357-3)
Supplement: Additional file 3: — Supplemental tables of assembly and scaffold statistics described in text. [file 12859_2014_357_MOESM3_ESM.docx]

**Additional file 3: Table 1.** Assembly statistics for samples produced by MDA on multiple cells (Controls)

| **Assembled After Preprocessing Step** | **Assembler** | **Contig Count** | **Contig N50** | **Len. of Longest Contig** | **Difference from Expected Genome Span (Ref) [Ħ]** | **Average Genome Coverage [Π]** | **Percent of Genome with >= 5x Coverage [ƍ]** | **0x Reference BPs (vs. Reads) [§]** | **0x Reference BPs (vs. Contigs) [¥]** | **Change in 0x Reference BPs (vs. Contigs) [¤]** |
| --- | --- | --- | --- | --- | --- | --- | --- | --- | --- | --- |
| ECOLI | G=4639754 |  |  |  |  |  |  |  |  |  |
| All Reads | Velvet-SC (K=55) | 525 | 24241 | 117731 | 27503 | 493.70 | 99.59 | 79809 | 322617 | -242808 |
| All Reads | IBDA-UD | 494 | 78996 | 209458 | 655151 | 897.71 | 97.34 | 79809 | 222959 | -143150 |
| All Reads | SPAdes | 531 | 95671 | 265,405 | 731025 | 888.04 | 97.09 | 79809 | 178557 | -98748 |
| Pre-Contam check | CLC | 661 | 22148 | 85370 | 223064 | 914.20 | 97.67 | 79804 | 247194 | -167390 |
| AllPaths Read Correct | CLC | 380 | 56893 | 178166 | 33975 | 856.78 | 99.62 | 164912 | 276055 | -111143 |
| DUST (Low Complexity Mask) | CLC | 372 | 54788 | 178160 | 23625 | 853.38 | 99.49 | 164766 | 271859 | -107093 |
| QV Trim | CLC | 491 | 28686 | 104172 | -28888 | 575.21 | 97.90 | 166089 | 272626 | -106537 |
| All preprocessing | Newbler | 1128 | 8614 | 60121 | -245550 | 501.61 | 98.69 | 166089 | 308970 | -142881 |
| All preprocessing | Velvet-SC (K=55) | 596 | 11576 | 59156 | -970734 | 375.08 | 99.80 | 166089 | 1029923 | -863834 |
| All preprocessing | IBDA-UD | 443 | 25968 | 77713 | -100013 | 586.13 | 98.93 | 166089 | 272651 | -106562 |
| All preprocessing | SPAdes | 412 | 38881 | 204,833 | -15751 | 575.38 | 98.78 | 166089 | 248626 | -82537 |
| RANDOM / RMKF Cutoff = 250 | Velvet-SC (K=35) | 559 | 14197 | 62611 | -191678 | 292.58 | 99.45 | 167697 | 393876 | -226179 |
| RANDOM / RMKF Cutoff = 250 | IBDA-UD | 460 | 30035 | 101797 | -10719 | 285.71 | 97.95 | 167697 | 276204 | -108507 |
| RANDOM / RMKF Cutoff = 250 | SPAdes | 416 | 33256 | 204,741 | 12278 | 283.66 | 98.20 | 167697 | 256895 | -89198 |
| RANDOM / RMKF Cutoff = 250 | Newbler | 880 | 10317 | 61260 | -134359 | 284.58 | 98.84 | 167697 | 287640 | -119943 |
| RANDOM / RMKF Cutoff = 100 | Velvet-SC (K=55) | 496 | 14710 | 59345 | -427867 | 140.27 | 99.81 | 168652 | 507003 | -338351 |
| RANDOM / RMKF Cutoff = 100 | IBDA-UD | 519 | 22982 | 89896 | -45023 | 136.17 | 98.15 | 168652 | 289490 | -120838 |
| RANDOM / RMKF Cutoff = 100 | SPAdes | 434 | 31949 | 105124 | -19371 | 135.13 | 98.26 | 168652 | 280220 | -111568 |
| RANDOM / RMKF Cutoff = 100 | Newbler | 673 | 14562 | 57100 | -110141 | 136.32 | 98.89 | 168652 | 284237 | -115585 |
| RANDOM / RMKF Cutoff = 80 | Velvet-SC (K=55) | 479 | 15232 | 49657 | -413641 | 116.62 | 99.82 | 169256 | 491568 | -322312 |
| RANDOM / RMKF Cutoff = 80 | IBDA-UD | 500 | 22905 | 62321 | -123092 | 114.64 | 98.89 | 169256 | 306377 | -137121 |
| RANDOM / RMKF Cutoff = 80 | SPAdes | 442 | 29712 | 204551 | -25399 | 112.07 | 98.27 | 169256 | 284905 | -115649 |
| RANDOM / RMKF Cutoff = 80 | Newbler | 600 | 17316 | 62787 | -107206 | 113.13 | 98.88 | 169256 | 283797 | -114541 |
| RANDOM / RMKF Cutoff = 40 | Newbler | 575 | 18263 | 62,787 | -112969 | 60.84 | 98.88 | 170175 | 282944 | -112769 |
| RANDOM / RMKF Cutoff = 40 | Velvet-SC (K=55) | 448 | 17038 | 67550 | -344007 | 62.68 | 99.77 | 170175 | 426956 | -256781 |
| RANDOM / RMKF Cutoff = 40 | IBDA-UD | 935 | 14477 | 62303 | -363447 | 61.59 | 98.88 | 170175 | 476670 | -306495 |
| RANDOM / RMKF Cutoff = 40 | SPAdes | 470 | 29712 | 105124 | -4818 | 60.27 | 98.30 | 170175 | 294391 | -294391 |
| TARGET FRAGMENTS / RMKF Cutoff = 40 | Newbler | 735 | 14387 | 57083 | -103319 | 85.09 | 98.67 | 170175 | 284549 | -114374 |
| TARGET FRAGMENTS / RMKF Cutoff = 40 | Velvet-SC (K=55) | 542 | 13676 | 61998 | -519303 | 88.19 | 99.80 | 170175 | 600814 | -430639 |
| TARGET FRAGMENTS / RMKF Cutoff = 40 | IBDA-UD | 482 | 28543 | 101797 | 794 | 85.01 | 97.72 | 170175 | 294791 | -124616 |
| TARGET FRAGMENTS / RMKF Cutoff = 40 | SPAdes | 448 | 31948 | 149929 | 36595 | - | - | 170175 | 269268 | -99093 |
| **S.AUREUS** | G=2872848 |  |  |  |  |  |  |  |  |  |
| All Reads | Velvet-SC (K=55) | 470 | 13968 | 53,575 | 17594 | 482.48 | 99.76 | 57823 | 47081 | 10742 |
| All Reads | IBDA-UD | 645 | 11522 | 61791 | -297356 | 32.74 | 96.23 | 57823 | 277069 | -219246 |
| All Reads | SPAdes | 677 | 10136 | 79523 | -284316 | 32.31 | 96.10 | 57823 | 195585 | -137762 |
| Pre-Contam check | CLC | 945 | 4815 | 32731 | -384898 | 31.34 | 95.97 | 57850 | 208435 | -150585 |
| AllPaths Read Correct | CLC | 782 | 6604 | 44774 | -615821 | 31.90 | 97.97 | 214657 | 210134 | 4523 |
| DUST (low complex) mask | CLC | 830 | 5466 | 44774 | -627194 | 31.52 | 97.56 | 217524 | 420387 | -202863 |
| QV Trim | CLC | 958 | 4234 | 21413 | -661450 | 24.04 | 96.16 | 230673 | 423806 | -193133 |
| All preprocessing | Newbler | 979 | 3768 | 23752 | -738329 | 24.31 | 97.00 | 230673 | 364319 | -133646 |
| All preprocessing | Velvet-SC (K=35) | 532 | 4193 | 22023 | -1303178 | 25.07 | 98.22 | 230673 | 1181416 | -966759 |
| All preprocessing | IBDA-UD | 866 | 4820 | 36989 | -703986 | 24.36 | 96.16 | 230673 | 627769 | -397096 |
| All preprocessing | SPAdes | 903 | 4795 | 58,232 | -600541 | 23.09 | 96.54 | 230673 | 455304 | -224631 |
| RANDOM / RMKF Cutoff = 80 | Newbler | 981 | 3768 | 23752 | -737824 | 24.25 | 96.99 | 230639 | 366860 | -136221 |
| RANDOM / RMKF Cutoff = 80 | Velvet-SC (K=35) | 532 | 4168 | 22023 | -1308264 | 25.04 | 98.22 | 230639 | 1184653 | -954014 |
| RANDOM / RMKF Cutoff = 80 | IBDA-UD | 867 | 4820 | 36989 | -703395 | 24.39 | 96.16 | 230639 | 628528 | -397889 |
| RANDOM / RMKF Cutoff = 40 | SPAdes | 904 | 4795 | 58232 | -599901 | 23.13 | 96.54 | 230639 | 455215 | -224576 |
| RANDOM / RMKF Cutoff = 40 | Newbler | 982 | 3768 | 23752 | -736651 | 23.96 | 96.98 | 230704 | 367324 | -136620 |
| RANDOM / RMKF Cutoff = 40 | Velvet-SC (K=35) | 531 | 4233 | 20520 | -1304708 | 25.04 | 98.21 | 230704 | 1180945 | -950241 |
| RANDOM / RMKF Cutoff = 40 | IBDA-UD | 865 | 4814 | 37001 | -599757 | 24.08 | 96.15 | 230704 | 627054 | -396350 |
| RANDOM / RMKF Cutoff = 40 | SPAdes | 904 | 4795 | 58232 | -599757 | 22.85 | 96.53 | 230704 | 455468 | -224764 |
| TARGETED / RMKF cutoff = 80 | Newbler | 980 | 3797 | 23752 | -737871 | 24.25 | 96.99 | 230662 | 366587 | -135925 |
| TARGETED / RMKF cutoff = 80 | Velvet-SC (K=35) | 528 | 4169 | 22023 | -1306125 | 25.06 | 98.20 | 230662 | 1185233 | -954571 |
| TARGETED / RMKF cutoff = 80 | IBDA-UD | 867 | 4820 | 36989 | -703476 | 24.37 | 96.16 | 230662 | 628689 | -398027 |
| TARGETED / RMKF cutoff = 80 | SPAdes | 904 | 4795 | 58,232 | -599901 | 23.13 | 96.53 | 230662 | 454789 | -224127 |
| TARGETED / RMKF cutoff = 80 | Newbler | 982 | 3694 | 23,752 | -737572 | 23.84 | 96.99 | 230629 | 364083 | -133454 |
| TARGETED / RMKF cutoff = 40 | Velvet-SC (K=35) | 532 | 4193 | 20520 | -1302512 | 24.93 | 98.22 | 230629 | 1183442 | -952813 |
| TARGETED / RMKF cutoff = 40 | IBDA-UD | 867 | 4820 | 36989 | -703251 | 23.95 | 96.16 | 230629 | 629044 | -398415 |
| TARGETED / RMKF cutoff = 40 | SPAdes | 905 | 4785 | 58232 | -599270 | 22.74 | 96.53 | 230629 | 454968 | -224339 |

Rapid CLC assemblies supply an indication of pre-processing effects on assembly span. Input for the NeatFreq coverage reduction software was post-automaton-only for samples E. coli and both post-automaton and all raw sequencing reads were tested for S. aureus. The latter sample’s top post-NeatFreq assemblies all resulted from the use of post-automaton input reads as expected when erroneous mers are not removed from the dataset. Of four Velvet-SC runs using K = (25,35,45,55), only the top result is shown. Top assemblies for each sample are highlighted as determined by fragmentation and greatest representation of its reference sequence (evaluated by aligning to the reference at 40% length and 90% identity cutoffs with contigs shredded to 7999 bp with 49 bp overlaps.). Contig N50 is calculated as the length of the contig for which a set of all consensus contigs of the same size or larger sums to at least half of the known reference span. Average contig coverage (Π) was calculated as a weighted mean of means across all contigs based on contig length. Low coverage reference bps represented in contigs (ƍ) are calculated using the alignment of input reads against the consensus that those reads produced. Annotated columns (Ħ, Π, ƍ, §, ¥, ¤) are highlighted for reference in text.

**Additional file 3: Table 2.** Assembly statistics for oversequenced phage (FR20V, BC391) and true single cell MDA (HMPMDA0100) samples

| **Assembled After Preprocessing Step** | **Assembler** | **Contig Count** | **Contig N50** | **Len. of Longest Contig** | **Difference from Expected Genome Span (Ref) [Ħ]** | **Average Genome Coverage [Π]** | **Percent of Genome with >= 5 X Coverage [**ƍ**]** | **0x Reference BPs (vs. Reads) [§]** | **0x Reference BPs (vs. Contigs) [¥]** | **Change in 0x Reference BPs (vs. Contigs) [¤]** |
| --- | --- | --- | --- | --- | --- | --- | --- | --- | --- | --- |
| **FR20V (fragment-only)** | G=37374 |  |  |  |  |  |  |  |  |  |
| All Reads | Newbler | 13 | 9296 | 11262 | 3004 | 476.55 | 93.57 | 0 | 224 | -216 |
| All Reads | Velvet-SC (K=35) | 18 | 671 | 1,240 | -24923 | 1564.09 | 94.78 | 0 | 7626 | 6929 |
| All Reads | IBDA-UD | 41 | 2507 | 4,410 | 15488 | 1110.58 | 72.73 | 0 | 416 | 183 |
| All Reads | SPAdes | 660 | 566 | 1,680 | 354134 | 167.25 | 9.71 | 0 | 62 | 0 |
| Pre-Contam Check | CLC | 128 | 906 | 10121 | 72657 | 274.30 | 55.90 | 0 | 478 | -478 |
| AllPaths Read Correct | CLC | 121 | 854 | 10852 | 68185 | 277.42 | 56.86 | 8 | 673 | -665 |
| DUST (Low Complexity Mask) | CLC | 123 | 863 | 10854 | 69538 | 280.00 | 56.58 | 8 | 551 | -543 |
| QV Trim | CLC | 122 | 829 | 10359 | 68197 | 278.33 | 55.93 | 8 | 461 | -453 |
| Contam check | CLC | 64 | 799 | 11534 | 34246 | 281.52 | 58.54 | 8 | 323 | -315 |
| Adapter Removal (ALL) | CLC | 30 | 775 | 11591 | 15518 | 385.77 | 73.60 | 8 | 170 | -162 |
| All Preprocessing | Velvet (K=25) | 18 | 689 | 1572 | -24414 | 35.14 | 41.80 | 8 | 2703 | -2695 |
| All Preprocessing | Newbler | 13 | 9296 | 11,262 | 3004 | 476.55 | 93.57 | 8 | 224 | -216 |
| All Preprocessing | Celera | 10 | 2918 | 9551 | -5960 | 378.98 | 97.65 | 8 | 5147 | -5139 |
| All Preprocessing | Velvet-SC (K=45) | 12 | 2752 | 6412 | -3203 | 732.26 | 99.94 | 8 | 124 | -1188 |
| All Preprocessing | SPAdes | 72 | 704 | 2503 | 18213 | 230.56 | 50.46 | 8 | 8 | -1188 |
| RANDOM / RMKF Cutoff = 120 | Newbler | 8 | 7982 | 11722 | -620 | 220.88 | 98.65 | 62 | 453 | -453 |
| RANDOM / RMKF Cutoff = 90 | Newbler | 7 | 7982 | 14473 | -351 | 176.12 | 98.66 | 108 | 457 | -457 |
| RANDOM / RMKF Cutoff = 60 | Newbler | 5 | 9384 | 14473 | -449 | 131.05 | 98.69 | N/A | 485 | -485 |
| RANDOM / RMKF Cutoff = 40 | Newbler | 7 | 9298 | 11565 | -642 | 96.92 | 98.45 | 145 | 576 | -576 |
| TARGETED / RMKF Cutoff = 60 | Newbler | 8 | 10350 | 11094 | -480 | 127.27 | 98.56 | 54 | 365 | -365 |
| TARGETED / RMKF Cutoff = 40 | Newbler | 5 | 9328 | 14,471 | -450 | 92.98 | 98.75 | 59 | 287 | -228 |
| TARGETED / RMKF Cutoff = 40 | Velvet-SC (K=35) | 4 | 24036 | 24036 | -476 | 93.14 | 98.45 | 59 | 165 | -8261 |
| BC391 (fragment-only) | G=13275 |  |  |  |  |  |  |  |  |  |
| All Reads | Newbler | 24 | 798 | 2,282 | 6286 | 270.26 | 75.69 | 0 | - | - |
| All Reads | Velvet-SC (K=45) | 13 | 1219 | 2,045 | 1039 | 1884.61 | 93.94 | 0 | 0 | 0 |
| All Reads | IBDA-UD | 106 | 532 | 2318 | 52339 | 586.59 | 68.52 | 0 | 0 | 0 |
| All Reads | SPAdes | 318 | 537 | 1348 | 166994 | 96.83 | 26.66 | 0 | 0 | 0 |
| Pre-Contam check | CLC | 39 | 571 | 2285 | 15034 | 798.05 | 60.75 | 0 | 0 | 0 |
| AllPaths Read Correct | CLC | 39 | 605 | 2270 | 16282 | 768.26 | 57.69 | 0 | 0 | 0 |
| DUST (Low Complexity Mask) | CLC | 39 | 605 | 2266 | 16328 | 767.08 | 57.75 | 0 | 0 | 0 |
| QV Trim | CLC | 40 | 605 | 2270 | 16831 | 754.21 | 57.62 | 0 | 0 | 0 |
| Contam check | CLC | 40 | 605 | 2270 | 16838 | 754.05 | 57.59 | 0 | 0 | 0 |
| Adapter Removal (ALL) | CLC | 26 | 965 | 2234 | 8601 | 1010.54 | 69.18 | 4 | 0 | 4 |
| All Preprocessing | Velvet | 3 | 523 | 530 | -11722 | 1.81 | 0.00 | 4 | 985 | -981 |
| All Preprocessing | Newbler | 13 | 1456 | 2,221 | 939 | 1532.55 | 92.64 | 4 | 104 | -100 |
| All Preprocessing | Celera | 1 | 1934 | 1934 | -11341 | 1365.47 | 99.48 | 4 |  |  |
| All Preprocessing | Velvet-SC (K=45) | 8 | 1334 | - | -3262 | 1914.23 | 99.90 | 4 | 0 | 4 |
| All Preprocessing | IBDA-UD | 1174 | 317 | - | 356562 | 75.13 | 35.92 | 4 |  |  |
| All Preprocessing | SPAdes | 26 | 854 | - | 7457 | 988.59 | 66.76 | 4 | 0 | 0 |
| RANDOM / RMKF Cutoff = 80 | Newbler | 12 | 1463 | 2,222 | -155 | 590.52 | 94.89 | 4 | 135 | -130 |
| RANDOM / RMKF Cutoff = 40 | Newbler | 12 | 1463 | 2,223 | 476 | 348.74 | 94.31 | 4 | 124 | -107 |
| TARGETED / RMKF Cutoff = 80 | Newbler | 11 | 1692 | 2,223 | -124 | 215.33 | 95.04 | 4 | 136 | -131 |
| TARGETED / RMKF Cutoff = 80 | Velvet (K=25) | 8 | 1738 | - | -1292 | 227.74 | 99.17 | 5 | 97 | -92 |
| TARGETED / RMKF Cutoff = 40 | Newbler | 12 | 1463 | 2,240 | 186 | 162.31 | 94.67 | 5 | 44 | -33 |
| **HMPMDA0100 (paired end** |  |  |  |  |  |  |  |  |  |  |
| All Reads | Celera | 174 | 6408 | 24107 | - | 188.15 | 98.12 | - | - | - |
| All Reads | Velvet-SC (K=55) | 95 | 17915 | 33,574 |  | 367.79 | 99.52 | - | - | - |
| All Reads | IBDA-UD | 204 | 16161 | 56201 |  | 303.08 | 91.21 | - | - | - |
| All Reads | SPAdes | 254 | 18768 | 53353 |  | 346.93 | 87.80 | - | - | - |
| Pre-Contam check | CLC | 231 | 15898 | 46352 | - | 282.81 | 90.69 | - | - | - |
| AllPaths Read Correct | CLC | 133 | 18788 | 40513 | - | 335.88 | 98.90 | - | - | - |
| DUST (Low Complexity Mask) | CLC | 172 | 11219 | 34546 | - | 318.93 | 97.66 | - | - | - |
| QV Trim | CLC | 341 | 3338 | 13813 | - | 250.77 | 94.88 | - | - | - |
| All Preprocessing | Velvet-SC (K=35) | 208 | 2847 | 11242 | - | 101.46 | 97.58 | - | - | - |
| All Preprocessing | IBDA-UD | 347 | 2972 | 13754 | - | 274.58 | 95.08 | - | - | - |
| All Preprocessing | SPAdes | 292 | 4534 | 27,109 | - | 285.88 | 96.08 | - | - | - |
| RANDOM / RMKF Cutoff = 40 | Newbler | 116 | 2664 | 6728 |  | 134.55 | 99.09 |  |  |  |
| RANDOM / RMKF Cutoff = 40 | Velvet-SC (K=55) | 53 | 2624 | 7417 |  | 140.42 | 99.54 | - | - | - |
| RANDOM / RMKF Cutoff = 40 | IBDA-UD | 113 | 2745 | 7505 |  | 135.74 | 96.41 | - | - | - |
| RANDOM / RMKF Cutoff = 40 | SPAdes | 88 | 4087 | 19453 |  | 131.58 | 98.26 | - | - | - |
| RANDOM / RMKF Cutoff = 40 | Newbler | 152 | 3089 | 11661 |  | 71.29 | 99.39 |  |  |  |
| RANDOM / RMKF Cutoff = 40 | Velvet-SC (K=55) | 106 | 2836 | 7048 |  | 72.49 | 99.30 | - | - | - |
| RANDOM / RMKF Cutoff = 40 | IBDA-UD | 164 | 2698 | 8136 |  | 72.09 | 96.96 | - | - | - |
| RANDOM / RMKF Cutoff = 40 | SPAdes | 120 | 5218 | 13665 |  | 70.35 | 99.02 | - | - | - |
| TARGETED / RMKF Cutoff = 80 | Newbler | 373 | 2580 | 13,767 |  | 69.70 | 97.18 |  |  |  |
| TARGETED / RMKF Cutoff = 80 | Velvet-SC (K=55) | 207 | 2838 | 12834 |  | 74.40 | 98.72 | - | - | - |
| TARGETED / RMKF Cutoff = 80 | IBDA-UD | 349 | 3028 | 13754 |  | 70.45 | 94.90 | - | - | - |
| TARGETED / RMKF Cutoff = 80 | SPAdes | 286 | 4534 | 28,437 |  | 66.96 | 95.66 | - | - | - |
| TARGETED / RMKF Cutoff = 40 | Newbler | 372 | 2632 | 13766 |  | 43.79 | 97.06 |  |  |  |
| TARGETED / RMKF Cutoff = 40 | Celera | 133 | 3956 | 13742 | - | 44.11 | 94.52 | - | - | - |
| TARGETED / RMKF Cutoff = 40 | Velvet-SC (K=55) | 207 | 2887 | 12834 |  | 48.16 | 98.71 | - | - | - |
| TARGETED / RMKF Cutoff = 40 | IBDA-UD | 350 | 2972 | 13743 |  | 44.18 | 94.76 | - | - | - |
| TARGETED / RMKF Cutoff = 40 | SPAdes | 291 | 4327 | 28437 |  | 42.29 | 95.52 | - | - | - |

Once again, only the top Velvet-SC run from K = (25,35,45,55) tests are shown. Sample HMPMDA0100 has no close reference so alignment-based calculations are not produced. Input for the NeatFreq coverage reduction software was post-automaton-only for samples FR20V and BC391 with post-automaton and all raw sequencing reads tested for HMPMDA0100. The latter sample’s top post-NeatFreq assemblies resulted from the use of post-automaton input reads as expected when erroneous mers are not removed from the dataset. When comparing missing reference bases for all samples except FR20V, there exists a case where the count of 0x regions in the dataset increased but the count in assembled reads decreases. In these cases, coverage reduction improves assembly by maintaining more reads required for a more contiguous Newbler assembly through low coverage regions. Targeted bin selection is shown to increase selection of true reference bases at low RMKF cutoff vs. the random equivalent. The high coverage HMPMDA0100 sample which caused Newbler to fail using all post-automaton input reads completes successfully at all reduced coverage levels. Top assemblies are highlighted, as determined by fragmentation and comparison to reference. Calculation methods for each column are available in the legend of Table 1. Annotated columns (Ħ, Π, ƍ, §, ¥, ¤) are highlighted for reference in text.

**Additional file 3: Table 3.** Scaffold statistics for all samples

| **Assembled after Pre-Processing Step** | **Assembler** | **Contig count** | **Contig n50** | **Total Contig Len. (Consensus Span)** | **Scaffold Count [α]** | **Scaffold N50 [β]** | **0x Reference BPs (vs. Contigs) [¥]** |
| --- | --- | --- | --- | --- | --- | --- | --- |
| **HMPMDA0100** | G=Unknown |  |  |  |  |  |  |
| All Reads | Celera | 174 | 6408 | 734101 | 174 | 6408 | - |
| All Reads | SPAdes | 254 | 18768 | 1033519 | 866 | 29776 | - |
| All Preprocessing | SPAdes | 292 | 4534 | 807558 | 385 | 6217 | - |
| RANDOM / RMKF Cutoff = 80 | Newbler | 116 | 2664 | 207295 | 20 | 16191 |  |
| RANDOM / RMKF Cutoff = 80 | SPAdes | 88 | 4087 | 218497 | 100 | 5307 | - |
| RANDOM / RMKF cutoff = 40 | SPAdes | 120 | 5218 | 341406 | 143 | 5711 | - |
| TARGETED / RMKF cutoff = 80 | Newbler | 373 | 2580 | 728275 | 81 | 16720 |  |
| TARGETED / RMKF cutoff = 80 | SPAdes | 286 | 4534 | 802949 | 425 | 6997 | - |
| TARGETED / RMKF cutoff = 80 | Newbler | 372 | 2632 | 731276 | 81 | 17629 |  |
| TARGETED / RMKF cutoff = 40 | Celera | 133 | 3956 | 404269 | 92 | 6469 | - |
| TARGETED / RMKF cutoff = 40 | SPAdes | 291 | 4327 | 793487 | 410 | 7499 | - |
| **E.COLI** | G=4639754 |  |  |  |  |  |  |
| All Reads | SPAdes | 531 | 95671 | 5370779 | 508 | 96035 | 178557 |
| All Pre-processing | Velvet | 2237 | 7905 | 4662775 | 221 | 43313 | N/A |
| All Pre-processing | Newbler | 1128 | 8614 | 4394204 | 221 | 44918 | 308970 |
| All Pre-processing | IBDA-UD | 443 | 25968 | 4539741 | 650 | 25968 | 272651 |
| All Pre-processing | SPAdes | 412 | 38881 | 4624003 | 283 | 97195 | 248626 |
| RANDOM / RMKF cutoff = 250 | IBDA-UD | 460 | 30035 | 4629035 | 595 | 57919 | 276204 |
| RANDOM / RMKF cutoff = 250 | SPAdes | 416 | 33256 | 4652032 | 296 | 87439 | 256895 |
| RANDOM / RMKF cutoff = 250 | Newbler | 880 | 10317 | 4505395 | 169 | 63944 | 287640 |
| RANDOM / RMKF cutoff = 100 | SPAdes | 434 | 31949 | 4620383 | 711 | 72097 | 280220 |
| RANDOM / RMKF cutoff = 100 | Newbler | 673 | 14562 | 4529613 | 136 | 62837 | 284237 |
| RANDOM / RMKF cutoff = 80 | SPAdes | 442 | 29712 | 4614355 | 721 | 95442 | 284905 |
| RANDOM / RMKF cutoff = 80 | Newbler | 600 | 17316 | 4532548 | 128 | 69899 | 283797 |
| RANDOM / RMKF cutoff = 40 | Newbler | 575 | 18263 | 4526785 | 134 | 63205 | 282944 |
| RANDOM / RMKF cutoff = 40 | SPAdes | 470 | 29712 | 4634936 | 788 | 60337 | 294391 |
| TARGET FRAGMENTS / RMKF cutoff = 40 | Newbler | 735 | 14387 | 4536435 | 142 | 69766 | 284549 |
| TARGET FRAGMENTS / RMKF cutoff = 40 | IBDA-UD | 482 | 28543 | 4640548 | 513 | 69466 | 294791 |
| TARGET FRAGMENTS / RMKF cutoff = 40 | SPAdes | 448 | 31948 | 4676349 | 762 | 90685 | 269268 |
| **S.AUREUS** | G=2872848 |  |  |  |  |  |  |
| All Reads | SPAdes | 677 | 10136 | 2588532 | 1110 | 11342 | 195585 |
| All Pre-processing | Newbler | 979 | 3768 | 2134519 | 311 | 3768 | 364319 |
| All Pre-processing | SPAdes | 903 | 4795 | 2272307 | 1738 | 9749 | 455304 |
| RANDOM / RMKF cutoff = 80 | Newbler | 981 | 3768 | 2135024 | 309 | 3768 | 366860 |
| RANDOM / RMKF cutoff = 80 | SPAdes | 904 | 4795 | 2272947 | 1739 | 9778 | 455215 |
| RANDOM / RMKF cutoff = 80 | Newbler | 982 | 3768 | 2136197 | 309 | 3768 | 367324 |
| RANDOM / RMKF cutoff = 40 | SPAdes | 904 | 4795 | 2273091 | 1738 | 9961 | 455468 |
| TARGET PAIRS / RMKF cutoff = 80 | Newbler | 980 | 3797 | 2134977 | 309 | 3797 | 366587 |
| TARGET PAIRS / RMKF cutoff = 80 | SPAdes | 904 | 4795 | 2272947 | 1739 | 9773 | 454789 |
| TARGET PAIRS / RMKF cutoff = 80 | Newbler | 982 | 3694 | 2135276 | 310 | 3694 | 364083 |
| TARGET PAIRS / RMKF cutoff = 40 | SPAdes | 905 | 4785 | 2273578 | 905 | 4785 | 454968 |

For the three test samples with avalable paired sequence, the top assembly is highlighted as determined by fragmentation and similarity to reference. The top scaffolding stats using sequencing data before reduction are highlighted by a dotted box andthe improved scaffold equivalent following reduction is highlighted in a solid box. All samples show improved scaffolding contiguity following pre-processing (E. coli - SPAdes) or pre-processing with coverage reduction (HMPMDA, S. Aureus - All Assemblers, E. coli - Newbler). Minimal change was seen in the scaffolds produced by IDBA-UD. Calculation methods for each column are available in the legend of Table 1. Annotated columns (α, β, ¥) are highlighted for reference in text.
